# Erratum

**DOI:** 10.1590/abd1806-4841.201893501

**Published:** 2020-12-05

In the manuscript “Solitary plantar basal cell carcinoma”, com número de DOI: 10.1590/abd1806-4841.20187079, published in the Anais Brasileiros de Dermatologia, 2018;93(3):419-21., in the page 421.

Where it reads:

“To date, only six patients with isolated BCC on the sole have been reported in the literature, by Rupec in 1982^5^, Ee *et al*. in 2004^8^, Gubianti *et al*.^7^ and Betti*et al*. in 2005^6^, Albedaño *et al*. in 2006^9^, and Ohata *et al*. in 2012.^10^”

“10. Ohata C, Imai N, Hinogami H, Akamatsu K, Sumimura Y. A case of basal cell carcinoma on the sole. J Cutan Pathol. 2012;39:56-62”

It should read:

“To date, only six patients with isolated BCC on the sole have been reported in the literature, by Rupec in 1982^5^, Ee *et al*. in 2004^8^, Gubianti *et al*.^7^ and Betti *et al*. in 2005^6^, Albedaño *et al*. in 2006^9^, and Sumimura et al. in 2012”

“10. Sumimura Y, Imai N, Akamatsu K,Hinogami H, Ohata C. A case of basal cell carcinoma on the sole. Skin Research. 2012;11:170-72.”

